# Coupled Metabolic Cycles Allow Out‐of‐Equilibrium Autopoietic Vesicle Replication

**DOI:** 10.1002/anie.202007302

**Published:** 2020-09-03

**Authors:** Anthonius H. J. Engwerda, Josh Southworth, Maria A. Lebedeva, Robert J. H. Scanes, Philipp Kukura, Stephen P. Fletcher

**Affiliations:** ^1^ Chemistry Research Laboratory University of Oxford 12 Mansfield Road Oxford UK

**Keywords:** autocatalysis, autopoiesis, metabolic network, out-of-equilibrium system, vesicles

## Abstract

We report chemically fuelled out‐of‐equilibrium self‐replicating vesicles based on surfactant formation. We studied the vesicles’ autocatalytic formation using UPLC to determine monomer concentration and interferometric scattering microscopy at the nanoparticle level. Unlike related reports of chemically fuelled self‐replicating micelles, our vesicular system was too stable to surfactant degradation to be maintained out of equilibrium. The introduction of a catalyst, which introduces a second catalytic cycle into the metabolic network, was used to close the first cycle. This shows how coupled catalytic cycles can create a metabolic network that allows the creation and perseverance of fuel‐driven, out‐of‐equilibrium self‐replicating vesicles.

## Graphical abstract

Continuous addition of a chemical fuel allows for the autocatalytic formation of surfactant that aggregate into vesicles. These vesicles can be maintained out‐of‐equilibrium by adding a catabolic catalyst to introduce a second, coupled reaction cycle into the metabolic network.

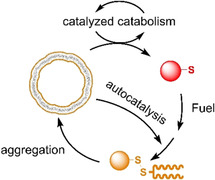

One of the great challenges in chemistry is to mimic the complex living structures found in nature by designing artificial out‐of‐equilibrium systems.^[1]^ These structures involve functional, dynamic states which require a continuous supply of energy to be sustained.^[2]^ This energy can be supplied as a chemical fuel, or another energy source such as light.^[3]^ Systems that use an energy supply to maintain behaviour that would otherwise not persist, are known as dissipative systems.^[4]^ Upon depletion or removal of the energy source, the rates of destruction and formation become unbalanced and the system moves to thermodynamic equilibrium. By maintaining complex systems in an out‐of‐equilibrium state different behaviour can be observed compared to systems in equilibrium. This includes novel self‐assembly processes,^[5]^ material properties^[6]^ and functionality.^[7]^

Out‐of‐equilibrium systems are frequently encountered in phase‐separated systems, where functionalities such as product selection^[8]^ or self‐regulation^[9]^ can be observed. Especially interesting is the creation of synthetic supramolecular vesicles, since these resemble cellular structures found in nature.^[10]^ Both functional transient vesicles,^[11]^ and thermodynamically stable vesicles exhibiting chemically fuelled functions have been explored.^[12]^ Alternatively, vesicles involving intricate reaction networks^[13]^ that can be compartmentalized enable functionality such as movement.^[14]^

Our group recently described how a system of self‐replicating surfactants that organized into micelles could be kept in an out‐of‐equilibrium state using a chemical fuel. Similar to a metabolic cycle, the continuous addition of a chemical fuel enables the metastable replicator to persist in a steady‐state (Figure 1 a).^[15]^ In that system a phase‐separated hydrophobic thiol **1** and aqueous disulfide **2** react, forming an amphiphilic molecule that self‐assembles into micellar aggregates. The aggregates catalyze their own formation, through a mechanism known as physical autocatalysis,[^16^, ^17^] where hydrophobic reactant is solubilized in the micelles. Such supramolecular systems, bounded from their environment and able to produce their own components by this mechanism of self‐replication are autopoietic species.^[18]^

**Figure 1:**
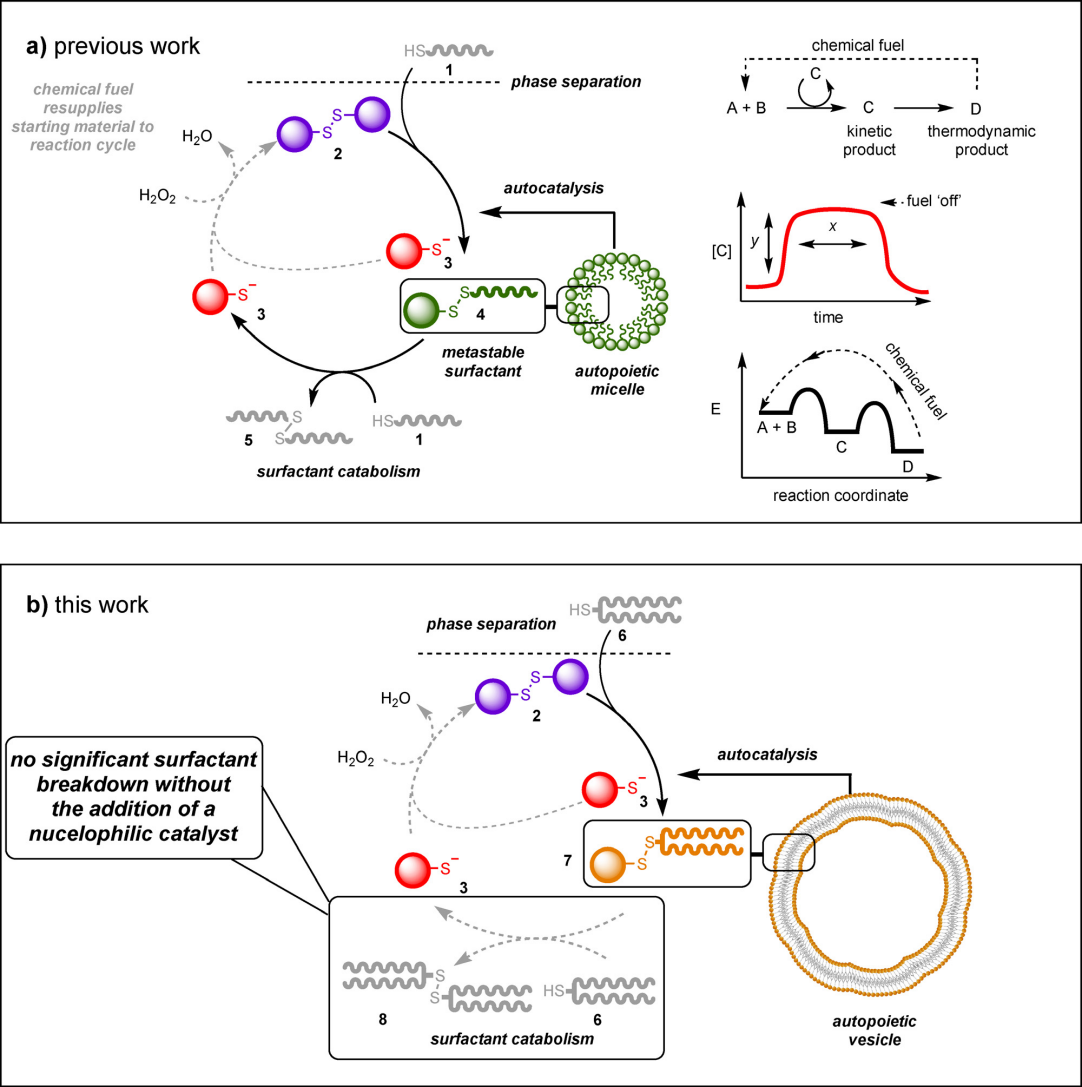
a) The previously investigated system of autopoietic micelles is based on the reaction between a phase‐separated thiol (**1**) and disulfide (**2**) to give a metastable surfactant (**4**) as the kinetic product. The kinetic product **4** breaks‐down upon reaction with an additional equivalent of thiol to form the thermodynamic product **3** and waste product **5**. By maintaining a continuous supply of chemical fuel (hydrogen peroxide), starting material **2** can be regenerated, thereby closing the metabolic cycle. Metastable surfactant **4** can be sustained in this situation, if thiol **1** is present, and the fuel supply is maintained. b) Intended catalytic cycle for the out‐of‐equilibrium formation of autopoietic vesicles composed of surfactant **7**. Under the used reaction conditions, degradation of **7** did not proceed to a sufficient extent, thereby impeding the closure of the catalytic cycle.

The micellar building blocks are transient electrophiles that undergo further reaction with excess thiol. The thermodynamic driving force of this reaction is the formation of waste product **3** as well as alkyl disulfide **5**. The surfactant can then be reformed by adding a continuous supply of chemical fuel (hydrogen peroxide), so that **3** is oxidized to regenerate disulfide **2**. This in turn can react with thiol **1** to allow for the continuous formation of the amphiphilic species **4**. Under these conditions, the rates of surfactant formation and destruction were balanced so that it could be held in an out‐of‐equilibrium state. By stopping the supply of chemical fuel, the amphiphile could no longer be regenerated, thereby returning the system to its thermodynamic equilibrium state.

Here, we report a system of autopoietic vesicles, and how they can be held in an out‐of‐equilibrium state. Such a vesicle‐based system was deemed to be one step closer to those found in nature as it would more closely mimic the boundaries of cells. We note that purely synthetic vesicle systems capable of self‐replication are underexplored compared to those based on micelles.^[17]^ In contrast to the linear surfactants used for the micelle‐forming systems, two‐tailed surfactants were used that form vesicles. Our initial studies on these more sterically encumbered surfactants showed that closing the metabolic cycle, necessary to maintain the system out‐of‐equilibrium was thwarted by these altered properties of the surfactant. This effectively stopped the catabolic step in the cycle and the system was merely autocatalytic in vesicle formation rather than being dissipative (Figure 1 b). For this reason, a catalyst for the catabolic step was added, effectively introducing a second, coupled catalytic cycle into the metabolic network.

In order to use the principles of the chemically fueled micelle system to design a system of autopoietic vesicles, we examined surfactants that might be predisposed to bilayer formation without disturbing key steps of the metabolic cycle. The cycle itself operates by a series of thermodynamically‐driven reactions that effect a unidirectional substrate flux as the system moves towards equilibrium. The system is maintained out‐of‐equilibrium by the selective oxidation step that closes the cycle. We envisioned that simply exchanging previously used 1‐octanethiol (**1**) to a more sterically hindered analogue would result in a larger molecular packing parameter, thereby favouring formation of vesicles.^[19]^ For this purpose, tridecane‐7‐thiol (**6**) was selected which possesses two hydrophobic tails and for which the corresponding surfactant (**7**) is reminiscent of the lipids found in cell‐membranes.^[20]^

We investigated the spontaneous formation of surfactant **7** from the reaction between phase‐separated disulfide **2** and thiol **6**. An initial lag‐period in the formation of **7** was followed by a sharp increase in concentration, giving rise to a sigmoidal curve typical of autocatalytic processes (Figure 2). Seeding experiments, in which a small quantity of **7** was added at the start of the experiment, resulted in elimination of the lag period, further consistent with autocatalysis.

**Figure 2:**
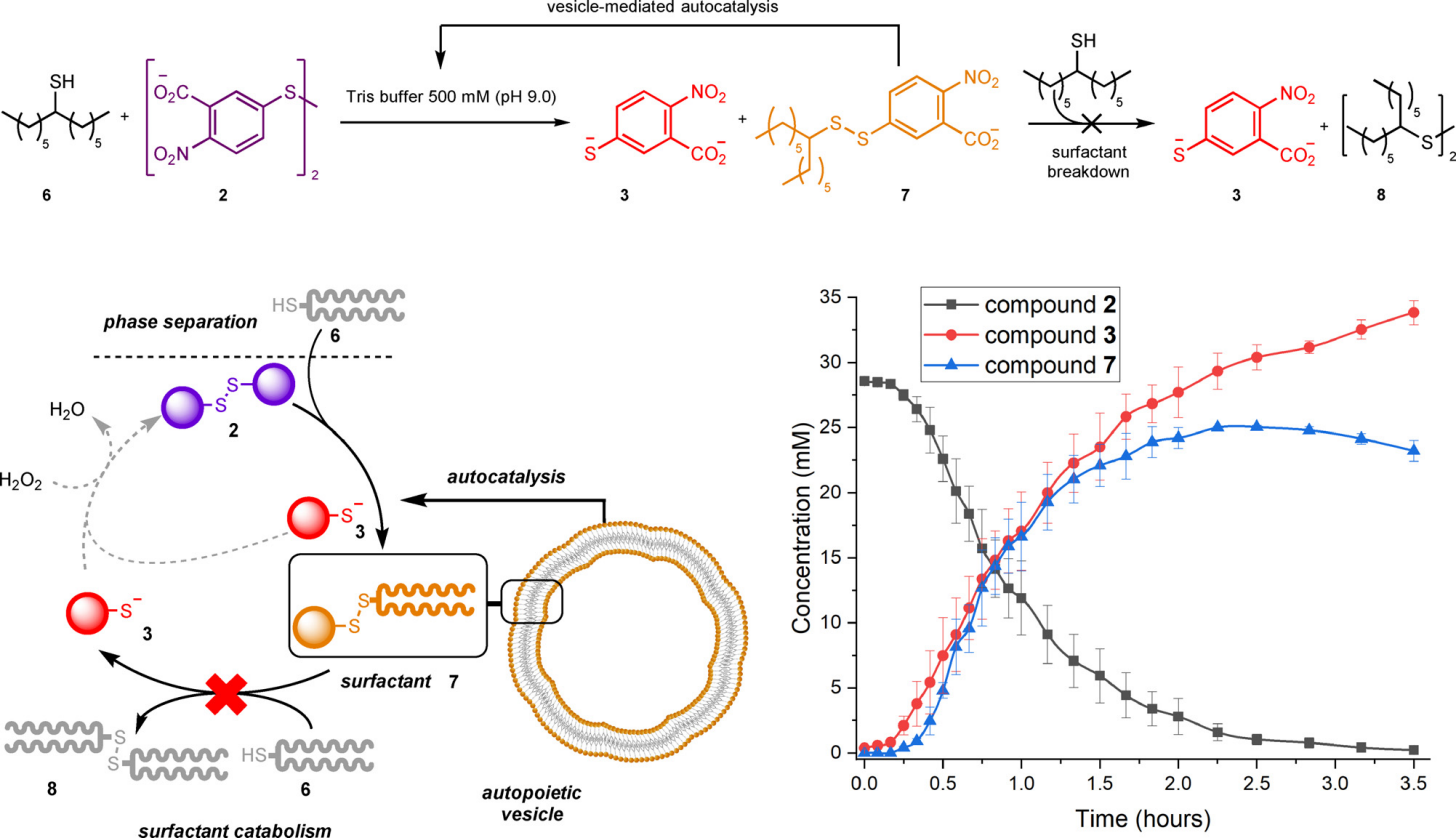
Intended reaction Scheme (top) and metabolic cycle (bottom left) for the out‐of‐equilibrium formation of autopoietic vesicles composed of surfactant **7**. Formation of vesicles proceeds autocatalytically, showing sigmoidal curves with respect to surfactant concentration. Under the reaction conditions the breakdown of **7** did not proceed to a sufficient extent to allow closure of the metabolic cycle.

Next, we studied the aggregation behaviour of **7**, to see whether this surfactant actually forms vesicles. Analysis of isolated **7** in water using Dynamic Light Scattering (DLS) pointed towards the formation of vesicles, showing an average aggregate size of around 150 nm (see Supporting Information). To better understand the nature and behaviour of these aggregates we used interferometric scattering microscopy (iSCAT). iSCAT allows visualization and quantification of nanoscale objects in solution by measuring changes in refractive index and the resulting scattering properties of the glass‐water interface when the aggregate particles bind non‐specifically to the glass surface.^[21]^ With iSCAT, the particle contrast is proportional to the mass for a variety of biological particles, such as proteins,^[22]^ molecular machines,^[23]^ antibodies,^[24]^ protein cages^[25]^ and virus capsids.^[26]^ At this stage, quantitative conversion of ratiometric contrast to particle size is not possible largely due to the lack of well‐characterized standards. Nevertheless, previous studies of lipid nanodiscs showed that changes in lipid mass can be accurately quantified.^[22]^ Making the approximation that lipids produce a similar contrast to peptides based on these results, the correlation between ratiometric contrast of a supramolecular aggregate and its mass allows us to estimate the number of monomers that form the aggregate particle, and therefore quantify particle size and growth.^[27]^

We have recently used iSCAT to investigate micelles and vesicles evolving in autocatalytic reactions either in situ on the microscope coverslip^[28]^ or under typical reaction flask conditions (by taking aliquots).^[27]^ Figure 3 a shows measurements of vesicles of **7** prepared by dissolving neat **7** in buffer. The aggregate particles appear as dark circles; the contrast of each particle is proportional to particle mass, and therefore, indirectly, to particle size. The corresponding contrast distribution histogram of **7** (Figure 3 b) is noticeably skewed, showing significant difference between the peak, median and mean contrast values (indicated by arrows on Figure 3 b). Such distribution is characteristic of vesicles, as compared to nearly symmetric distributions with much lower contrast we have previously observed for micelles (Supporting Information Table S5.1).^[27]^ Significant differences in the peak, median and mean of the distribution suggest that the aggregate population is polydisperse, and there is substantial variation in the size of the particles that are present in solution. Our observation of a highly polydisperse mixture was confirmed by DLS measurements, showing a significantly broadened size distribution.

**Figure 3:**
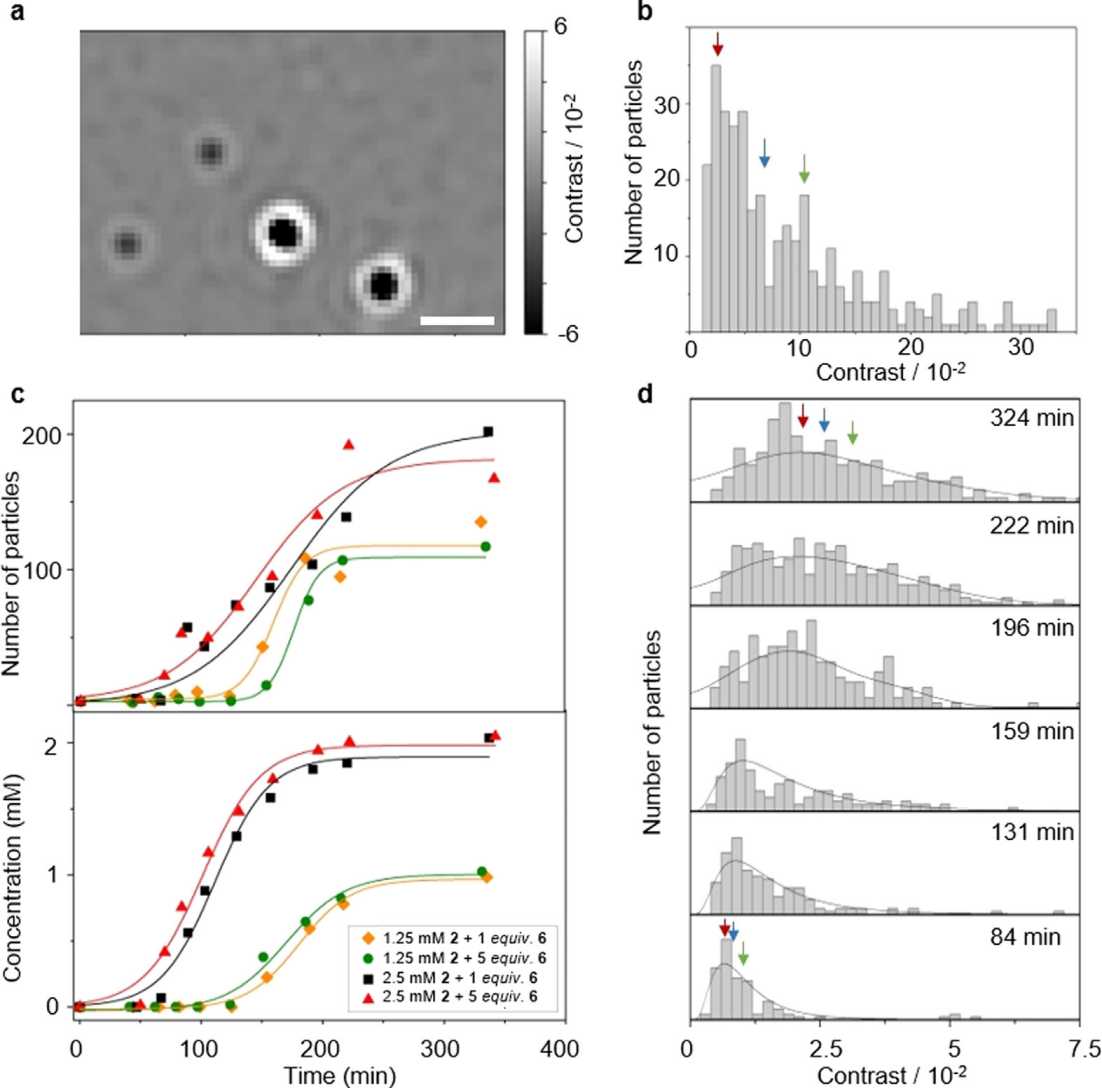
iSCAT characterisation of the aggregates of **7**. a) iSCAT image of aggregates of **7**; scale bar 1 μm; b) Ratiometric contrast histogram obtained from imaging a 200 μM solution of **7** in 0.5 m TRIS buffer at pH 9; arrows indicate the peak (red), median (blue) and mean (green) contrast; c) characterisation of reaction kinetics in biphasic reactions between **6** and **2** under varied reaction conditions: by counting the aggregate particles of **7** by iSCAT (top) and by monitoring the concentration of **7** by UPLC (bottom); iSCAT data fitted into the Hill equation, showing a sigmoidal profile in each case; d) changes in the ratiometric contrast distribution over the course of a biphasic reaction between 2.5 mm **2** and 5 equiv of **6**.

iSCAT also allows real‐time monitoring of the aggregates of **7** as they evolve in biphasic reactions between **6** and **2**. In order to study the kinetics of the formation of the aggregates by iSCAT, we have taken aliquots throughout the reaction, and counted the number of particles binding to the glass in each aliquot.^[27]^ All experiments showed a sigmoidal profile, with a clear lag period when no particles were detected, followed by growth in the population of aggregates (Figure 2 c, top). These results are in good agreement with those obtained by following the concentration of **7** by UPLC (Figure 3 c, bottom), suggesting that the aggregates form as soon as detectable amount of surfactant is present. The formation of these aggregates is not limited by the availability of thiol, since increasing the amount of **6** from 1 to 5 equivalents has no marked effect on the concentration profile of **7**. Figure 3 d shows the changes in the contrast distribution over the course of a biphasic reaction between **2** and **6**. It is evident that the contrast, and therefore particle size, increases throughout the reaction. The particles that emerge just after the end of the lag period are small, with a contrast similar to what we observed previously for micelles (Figure 3 d bottom and Table S5.1).^[27]^ As the reaction progresses, the peak contrast increases up to 3 times and becomes comparable to that observed for the solution of vesicles of **7**, suggesting formation of small vesicles. Notably, the contrast distribution of these particles is more symmetric. Thus, near the completion point (Figure 3 d top), the peak, median and mean values are quite similar, unlike what we observed for a solution of **7** obtained by dissolution of neat surfactant (Supporting Information Table S5.1). Figure S5.1 (see Supporting Information) shows changes of the mean, median and peak contrast under varied concentrations of the reagents as the reaction progresses. In all cases, a gradual increase is observed, implying growth of aggregate size over the course of the reaction. The growth is faster in the presence of the excess of thiol, however the final particle size and polydispersity is mostly independent of the reaction conditions. Thus, the mean, median and peak contrast remain similar even after 20 h. This suggests that the vesicles evolving in the reaction are relatively small and uniform, unlike when neat surfactant is dissolved in buffer.

The breakdown of previously reported surfactant **4** proceeded swiftly at room temperature, resulting in the formation of waste‐product **5**. However, for the branched surfactant **7** studied here, a degradation rate adequate for keeping the system out‐of‐equilibrium was not observed. By using an elevated reaction temperature (40 °C), and additional equivalents of thiol (7 equiv.) the half‐life of **7** was still well over 4 hours (see Supporting Information). It is likely that the increased steric bulk of both surfactant and the thiol, combined with the lower solubility of the thiol results in a slow catabolic process.^[29]^ To create an out‐of‐equilibrium system, continuous formation and destruction of **7** are required here. We envisioned that the breakdown of **7** could be promoted by a nucleophilic catalyst,^[30]^ thereby creating a second, coupled metabolic cycle (Figure 4 a). The catalyst, acting as a nucleophile should form adduct **10** by displacement of the leaving group. Compound **10** is itself a transient species which undergoes further reaction with thiol **6** to give the di‐alkyldisulfide **8** and regenerate the catalyst **9**. The ideal catalyst therefore needs to be both a good nucleophile and leaving group, while being compatible with the rest of the system. To this end, we found DMAP (**9**), a widely used nucleophilic catalyst in synthesis, proved effective for the degradation of **7**. Addition of 0.25 equivalents of DMAP to a mixture of surfactant **7** and thiol **6** (6 equivalents) at 40 °C reduced the half‐life of **7** to just 40 minutes, compared to over 4 hours in the absence of DMAP (Figure S2.1). We found that when 0.25 equivalent of DMAP was added at the start of the biphasic reaction involving **6** and **2** that the lag period and subsequent increase in the concentration of **7** were quickly followed by a decrease in [**7**] (Figure 4 b). In these experiments the surfactant has become a transient species that is no longer formed upon depletion of **2**.

**Figure 4:**
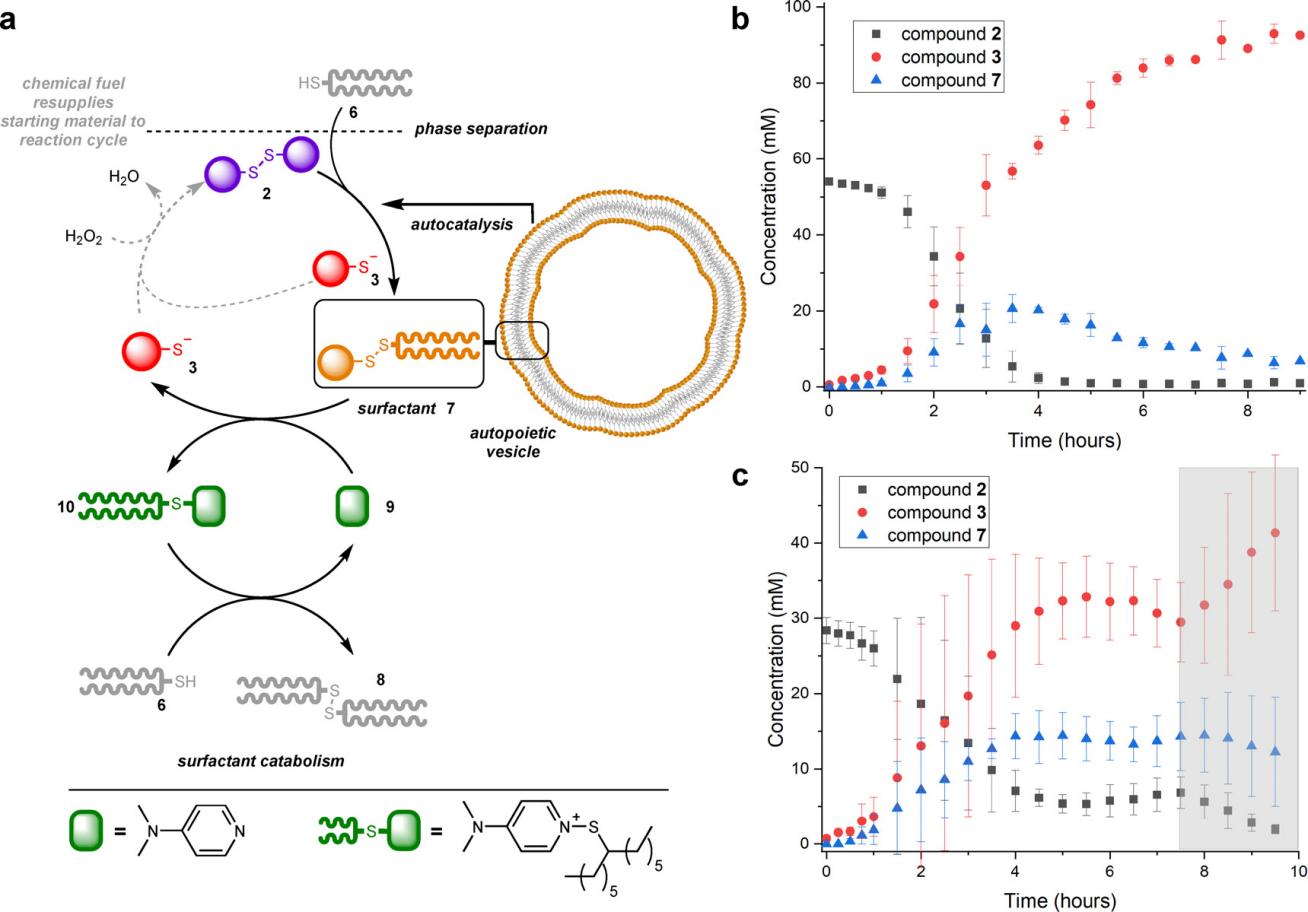
a) Introduction of a second metabolic cycle allows transient formation of vesicles composed of **7**. DMAP (**9**) acts as a nucleophilic catalyst to destroy **7**, thereby closing the original metabolic cycle. b) Biphasic reaction of disulfide **2** and thiol **6** in the presence of 0.25 equivalent DMAP, which catalyzes the breakdown of **7**, so that it becomes transient. c) Continuous addition of hydrogen peroxide as a chemical fuel to the autopoietic system closes the metabolic cycle and maintains the system in an out‐of‐equilibrium state. Discontinuation of the chemical fuel (grey marked area), results in the system moving to thermodynamic equilibrium. No surfactant **7** could be detected 12 hours after switching off the peroxide flow.

To maintain the concentration of **7** in an out‐of‐equilibrium state, both continuous synthesis and anti‐synthesis of **7** is required. Since the breakdown of **7** can be realized using the nucleophilic catalyst, regeneration of **2** is required to close the original substrate cycle (Figure 4 a). Oxidation of **3** was achieved via syringe pump addition of hydrogen peroxide to provide a continuous supply of **2**, which allowed vesicles composed of **7** to be sustained (Figure 4 c). Akin to living systems, discontinuation of the fuel does not result in the immediate death, but rather a slow decay of the system. When the supply of chemical fuel is halted (or, at longer times, thiol **6** gets depleted) no more surfactant can be formed. In this situation, surfactant formation is no longer balanced by its breakdown, resulting in a decrease of concentration over time. This results in the slow disappearance of the transient vesicles, returning the system to thermodynamic equilibrium. The thermodynamic driving force for the overall process is the conversion of high energy disulfide **2** and thiol **6** into disulfide **8** and thiol **3**. The electron withdrawing groups on **3** stabilizes the negative charge of this waste product in the basic buffer. Energy dissipation in the reaction cycle makes it irreversible, giving directionality to the metabolic cycle.

Increasingly chemistry is focused on designing and operating out‐of‐equilibrium systems, as these can achieve functions not possible under thermodynamic equilibrium. These goals are relevant to prebiotic chemistry, one of the aims of that field is to learn how primitive cells could form under abiotic conditions.^[31]^ In this work, we established how spontaneously emerging vesicles could be kept in life‐like states by coupling two reaction cycles. The emergence of these vesicles was studied by iSCAT, which showed initial autocatalytic growth in the number of the aggregates, and that vesicles’ average size also increased over time. By continuously supplying chemical fuel, the rates of formation and breakdown of these primitive cells can be balanced allowing their out‐of‐equilibrium state to be sustained via the dissipation of energy through the two catalytic cycles. This work may provide direction for the development of primitive synthetic lifeforms, using coupled metabolic pathways to sustain dissipative supramolecular structures.

## Conflict of interest

The authors declare no conflict of interest.

## Supporting information

As a service to our authors and readers, this journal provides supporting information supplied by the authors. Such materials are peer reviewed and may be re‐organized for online delivery, but are not copy‐edited or typeset. Technical support issues arising from supporting information (other than missing files) should be addressed to the authors.

Supplementary

Supplementary

Supplementary
