# Supplementary material for: Coupled Metabolic Cycles Allow Out‐of‐Equilibrium Autopoietic Vesicle Replication
Source: Angew Chem Int Ed Engl. 2020 Sep 3;59(46):20361–6. doi: 10.1002/anie.202007302 (PMC7692917; doi:10.1002/anie.202007302)
Supplement: Supplementary file 1 — Supplementary [file ANIE-59-20361-s001.pdf]

Angewandte Chemie
Eine Zeitschrift der Gesellschaft Deutscher Chemiker

Supporting Information

Coupled Metabolic Cycles Allow Out-of-Equilibrium Autopoietic Vesicle Replication

Anthonius H. J. Engwerda, Josh Southworth, Maria A. Lebedeva, Robert J. H. Scanes, Philipp Kukura, and Stephen P. Fletcher**

anie_202007302_sm_miscellaneous_information.pdf
anie_202007302_sm_Video1.mp4
anie_202007302_sm_Video2.mp4

Supplemental Information

1	Biphasic experiments	2
2	Destruction experiments	3
3	Ring tensiometry data	4
4	DLS measurements	5
5	iSCAT measurements	6
6	Synthesis of compounds	10
7	UPLC calibration	13
8	NMR spectra	14
9	References	19

1) Biphasic experiments:

Surfactant formation:

In a typical experiment, a total of 23 mg compound **2** (0.058 mmol), 2 mL TRIS buffer (0.5M, pH 9.00) and an oval stirring magnet (1.0 by 0.5 cm) were added to a 10 mL roundbottom flask. The solution was stirred at 450 rpm and the vial heated to 40 ˚C. After compound **2** had completely dissolved, 100 µl compound **6** (0.42 mmol) was carefully added on top of the water layer and stirring was continued.

Catalysed destruction experiments:

In a typical experiment, 40 mg compound **2** (0.10 mmol), 3 mg DMAP (0.025 mmol), 2 mL, TRIS buffer (0.5M, pH 9.00) and a oval stirring magnet (1.0 by 0.5 cm) were added to a 10 mL roundbottom flask. The solutions were stirred at 450 rpm and the vial was heated to 40 ˚C. After compound **2** had completely dissolved, 100 µl compound **6** (0.42 mmol) was carefully added on top of the water layer while stirring was continued.

Chemically fuelled experiments:

In a typical experiment, 23 mg compound **2** (0.058 mmol), 3 mg DMAP, 2 mL, TRIS buffer (0.5M, pH 9.00) and a oval stirring magnet (1.0 by 0.5 cm) were added to a 10 mL roundbottom flask. The solutions were stirred at 450 rpm and the vial was heated to 40 ˚C. After compound **2** had completely dissolved, 100 µl compound **6** (0.42 mmol, 7 equiv.) was carefully added on top of the water layer and the addition of hydrogenperoxide (1.5 M, 10 µL/h) was started (while stirring).

Sampling:

Samples were taking regularly, by carefully extracting 20 µL of the aqeous layer. The extracted solution was immediately added to 1 mL aqeous solution of maleimide (62 mM, containing 0.16 mM 3-methyl-2-nitrobenzoic acid as an internal reference) to quench all remaining thiols. Samples were analyzed as described in the UPLC section.

2) Destruction experiments:

For the destruction experiments, 20 mg of **7** and 60 mg **6** (6 equiv.) were added to 2 mL TRIS buffer (0.5 M, pH 9.00) in the presence or absence of 1.6 mg DMAP (0.25 equiv.). The conversion of **7** to NTB (**3**) was monitored over time using UPLC (Fig S2.1). The presence of 0.25 equiv. of DMAP increased the rate of destruction of **7** by a factor of 6.

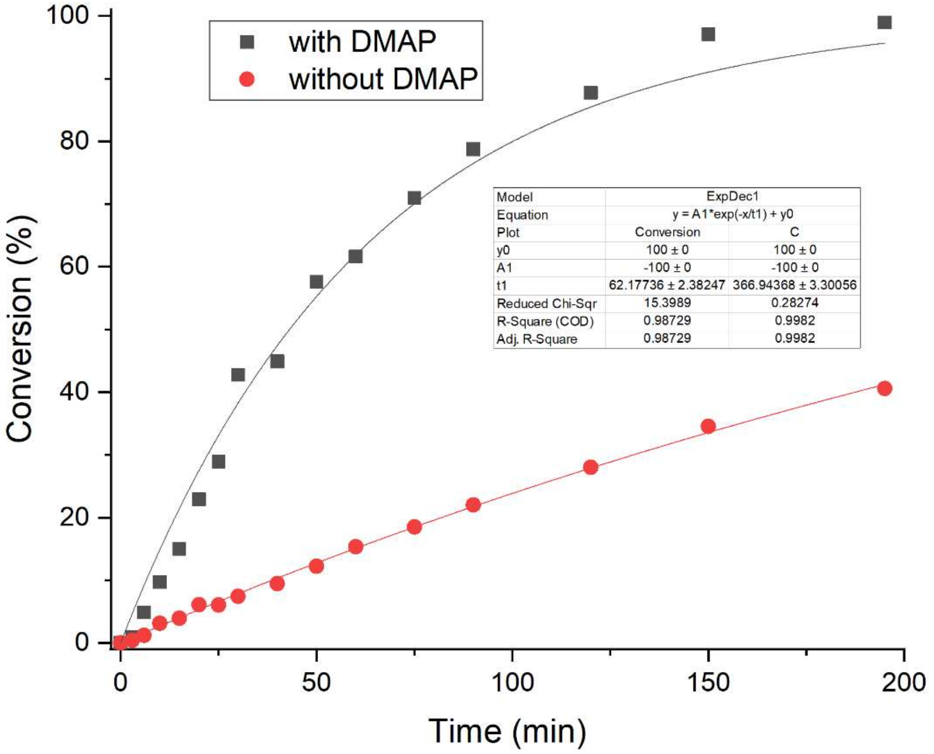

Figure S2.1: Destruction of compound **7** in TRIS buffer (at 40 ˚C) the presence and absence of DMAP as a catalyst.

3) Ring tensiometry data:

Surface tension measurements were made by the du Noüy ring method and used to calculate the critical aggregation concentration (CAC) of compound **7**. Surface tension is plotted against ln[**7**], whereby the point from which the surface tension no longer decreases corresponds with the CAC (Fig. S3.1). This can be calculated using the intercept of the two drawn lines.

$$Critical\ Aggregation\ Concentration\ (CAC) = 0.058\ mM$$

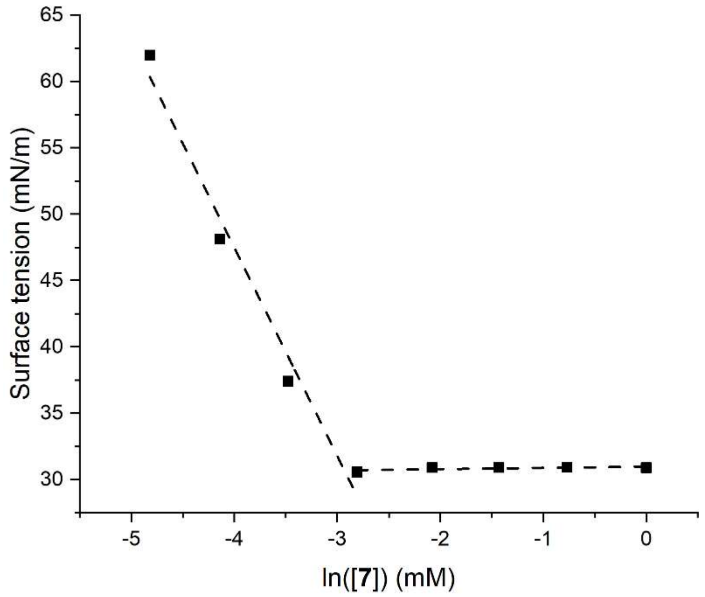

Figure S3.1: Plot of the surface tension versus ln([**7**]) in a TRIS buffer (0.5 M, pH 9.00). The intercept of the two lines corresponds to the critcal aggregation concentration (CAC).

4) DLS measurements:

Analyses were performed using a Malvern Zetasizer Nano ZEN5600 recording particle and molecule size. Instrument control and data processing were performed using Zetasizer software. Disposable plastic cuvettes were used with 1.0 mL of sample solution. Measurements were performed thrice for every concentration. Measurements were made using an equilibrated heating probe at 60 °C, setting the appropriate parameters for water. All samples were prepared in a TRIS buffer (0.5 mM, pH 9.00) and were filtered using a microfilter (poresize 0.22 μm) prior to measurement.

Measurements on solution containing compound **7** gave varying maximum intensities of the size distribution depending on the concentration (Fig. S4.2).

			Size (d.n...	% Intensity:	St Dev (d.n...
Z-Average (d.nm):	139.0	Peak 1:	163.2	100.0	68.51
PdI:	0.139	Peak 2:	0.000	0.0	0.000
Intercept:	0.969	Peak 3:	0.000	0.0	0.000
Result quality	Good				

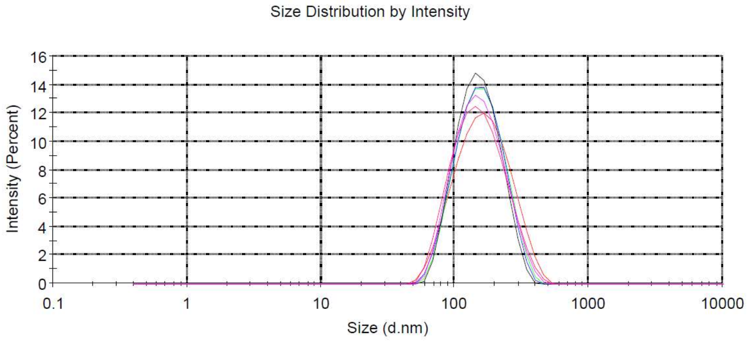

Figure S4.1: DLS measurement on a 4 mM solution of compound **7** at pH 9.00.

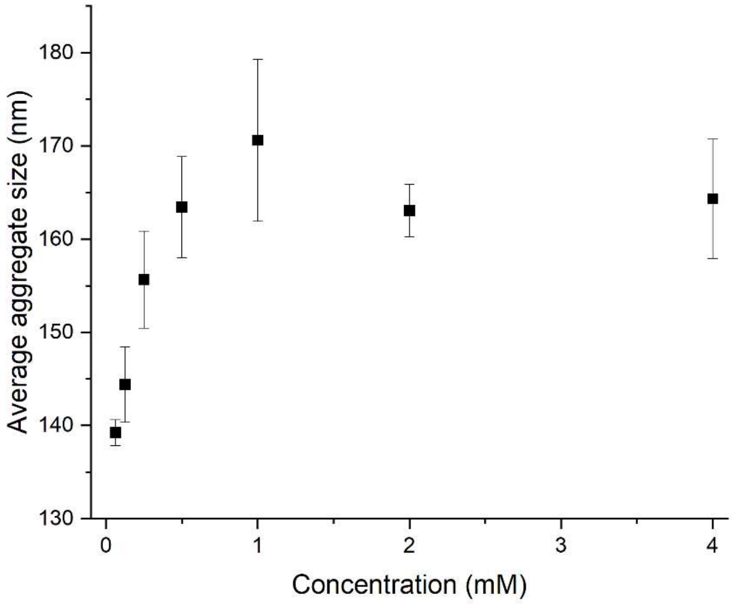

Figure S4.2: Average aggregate size for various concentrations of compound **7** at pH 9.00. Error bars correspond to the standard deviation of the means of three experiments.

5) iSCAT measurements:

Biphasic reactions between 1 and 2 investigated by iSCAT:

A 5 mM stock solution of **2** in TRIS buffer was prepared by dissolving **2** (19.8 mg, 0.05 mmol) in 10 mL TRIS buffer (0.5 M, pH 9.00). The solution was briefly sonicated to break any remaining undissolved particles, filtered through a microfilter (poresize 0.22 μm) and stored at 4 ˚ C.

In a typical biphasic reaction, an aliquot of stock solution was placed in a 10 mL round bottom flask (RBF) and diluted with TRIS buffer (0.5 M, pH 9.00) to 2.5 mM or 1.25 mM concentrations, to make 2 mL solutions of **2**. Thiol **6** (1 equiv. or 5 equiv.) was then carefully added to the top of the aqueous solution, and the reaction mixture was stirred at 450 rpm. At given time intervals, 0.5 μL aliquots were taken from the aqueous layer, diluted with 49.5 μL of buffer (100-fold dilution), and 30 s iSCAT videos were recorded immediately. At the same time, 20 μL aliquots were taken for UPLC analysis.

iSCAT Setup:

The iSCAT experimental set-up is similar to that described by Young et al,[1] with a 520 nm diode laser used as the incident light source. Frames were recorded at 1 kHz with an exposure time of 0.98 ms, using a CMOS camera. Focus in the z axis is maintained using an autofocus system relying on the total internal reflection (TIRF) of a 638 nm beam. Instrument control was performed using the custom software written in LabView.

Data processing:

Data processing was performed using the custom software written in Python, as described elsewhere. In brief, differential imaging was achieved by subtracting sets of images temporally offset by a time Δt. The signal-to-noise ratio was then improved by spatially (3 x 3 binning) and temporally averaging the differential images (15 images).

Particle detection was performed as described by Young et al.[1] Briefly, diffraction-limited spots were identified by the software, and fitted to the 2D Gaussian function to give the ratiometric contrast value. The possibility of each diffraction limited spot being attributed to more than one particle was excluded by considerations of landing rates similar to those described by Ortega-Arroyo et al.[2] In each 30 s video, the number of fitted particles corresponding to binding events was counted to quantify reaction kinetics.

Coverslips and sample preparation for iSCAT analysis:

Samples for iSCAT analysis were prepared using ultrapure MilliQ water and filtered through 0.2 μm cellulose filters. Reaction mixture aliquots were analysed as obtained, without further purification.

Glass coverslips (no. 1.5, 24 x 50 mm, VWR; and 24 x 24 mm, VWR) were cleaned by sequential sonication in MilliQ water, isopropanol and MilliQ water (5 min each), and dried under the stream of nitrogen. Clean coverslips were assembled into flow chambers using double-sided-sticky tape (3M) as described by Young et al.[1] Fresh aluminium foil was folded

around an A4 size cutting board. Individual 24×24 coverslips were taped using two strips of double-sided tape and cut from the foil using a scalpel blade. Each excised 24×24 coverslip was joined, tape side down, in the centre of a 24×50 coverslip and stored prior to use.

Table S5.1: Summary of the ratiometric contrast of the vesicles of **7** and micelles of **4** measured by iSCAT. Peak contrast is obtained from multiple gaussian fit of the contrast distribution in Python; median and mean contrast calculated for each distribution in Python.

Sample	Peak contrast	Median contrast	Mean contrast
Vesicles of pure **7**	0.029±0.006	0.065±0.007	0.10±0.01
Micelles of pure **4**	0.0067±0.0009	0.0076±0.001	0.010±0.003
Reaction between 2.5 mM **2** and 5 *equiv*. **6** after 1.5 h	0.0075	0.0081	0.012
Reaction between 2.5 mM **2** and 5 *equiv*. **6** after 6 h	0.021	0.025	0.030

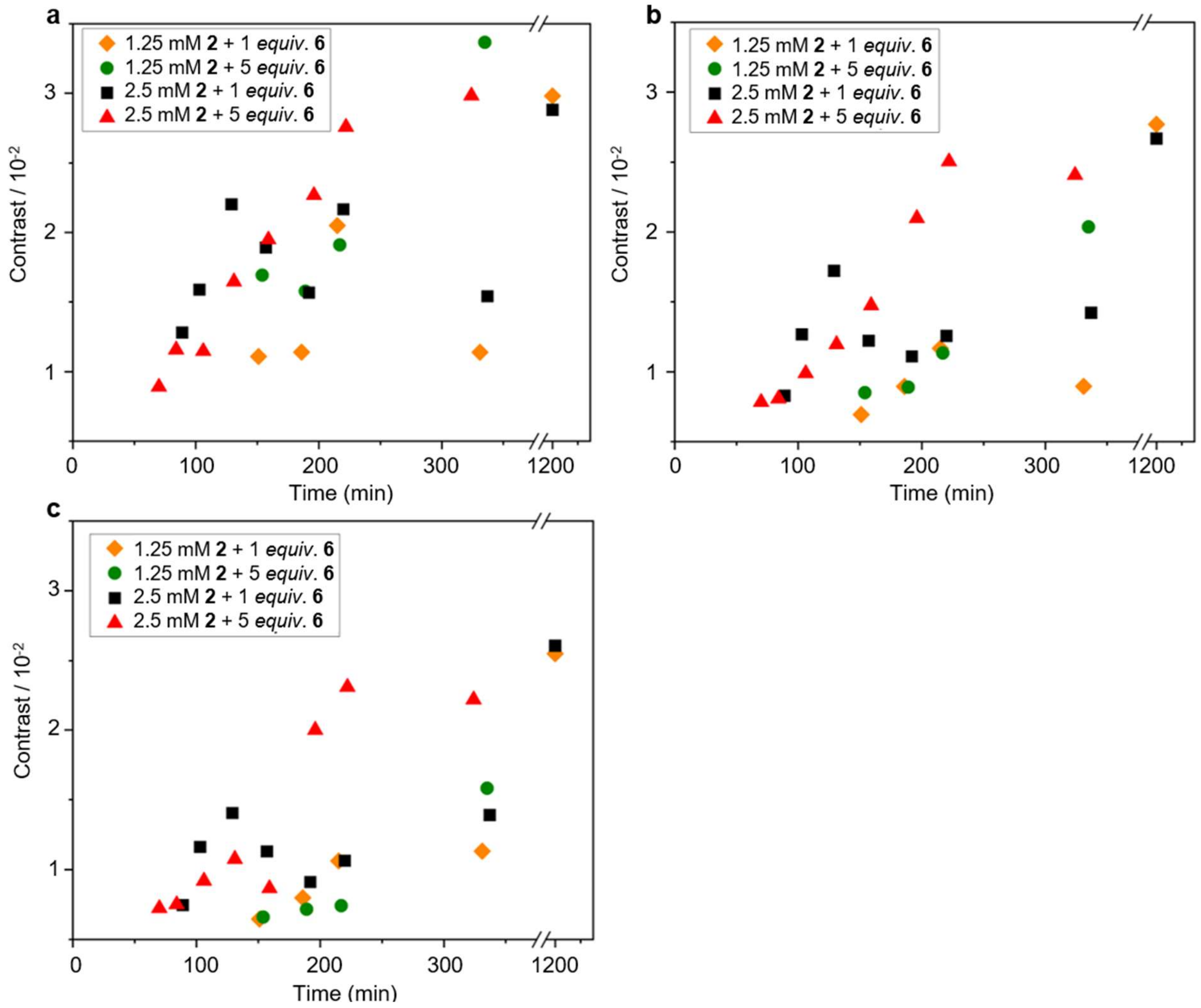

Figure S5.1: Changes in a) mean, b) median and c) peak contrast over the course of biphasic reactions between **6** and **2** under varied concentrations of reagents.

Correlation between iSCAT ratiometric contrast and mass:

The results of Fig S5.2 and table S5.3 show that the iSCAT contrast increases with the increasing hydrodynamic radius of a particle. This correlation is however non-linear and quantifying this relationship will require creating a substantial library of calibrants which are not readily available.

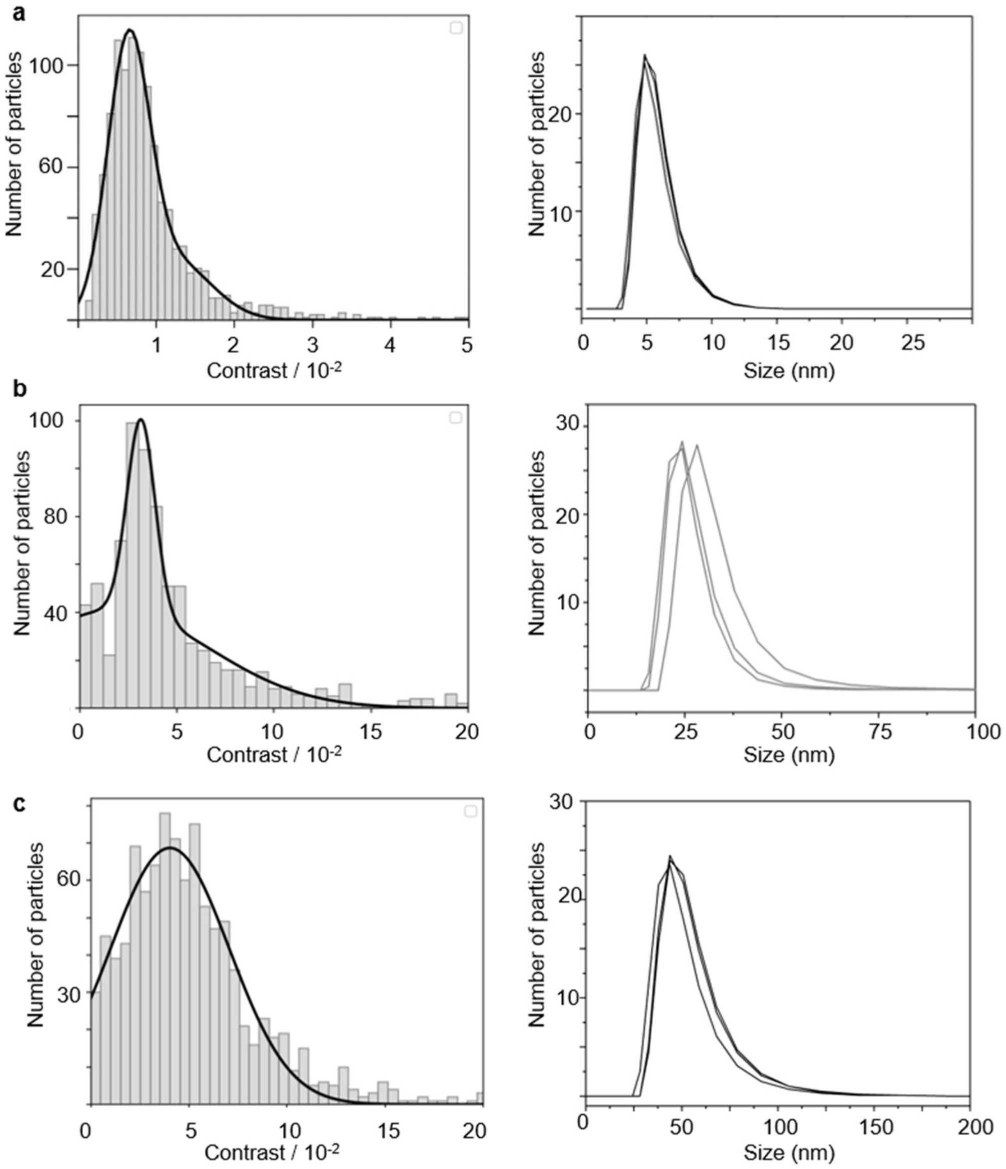

Figure S5.2: iSCAT contrast distribution histograms (left) and DLS number average distributions (right) for different aggregates: a) micelles of lipid **4**; b) sonicated unilamellar DOPC vesicles; c) vesicles of **10**.

Table S5.3: Correlation between iSCAT contrast and DLS number average distribution for different lipid aggregates.

Compound	Mean iSCAT contrast	Hydrodynamic radius, nm	Ref
**4**	0.01	6	[2]
Sonicated unilamellar DOPC vesicles	0.045	24	[2]
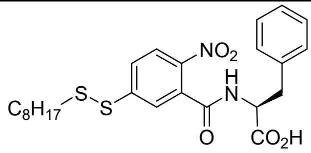 , **10**	0.065	40	[2]
**7**	0.1	150	This work

Supporting videos.

Video 1. Vesicles of **7** made by dissolving neat surfactant in buffer.

Video 2. Vesicles of **7** evolved in the reaction between 2.5 mM **2** and 1 equiv. of **6** after 22 h (at the completion point).

6) Synthesis of compounds:

All commercial chemicals were purchased from Sigma-Aldrich and were used without further purification. Reactions were followed using thin layer chromatography (TLC) on silica gel-coated plates (Merck 60 F254). Detection was performed with UV-light (254 nm), and/or by charring at ~150 °C after dipping into a solution of $KMnO_4$ (1 g/100 mL in ethanol). NMR spectra were recorded on a Bruker 400 (400 MHz) spectrometer in $CDCl_3$ (unless otherwise reported). Chemical shifts are given in ppm with respect to tetramethylsilane (TMS) as internal standard. Coupling constants are reported as *J*-values in Hz. Column chromatography was carried out using Acros silica gel (43-60 µm). Compound **2** was synthesised according to scheme 6.1.

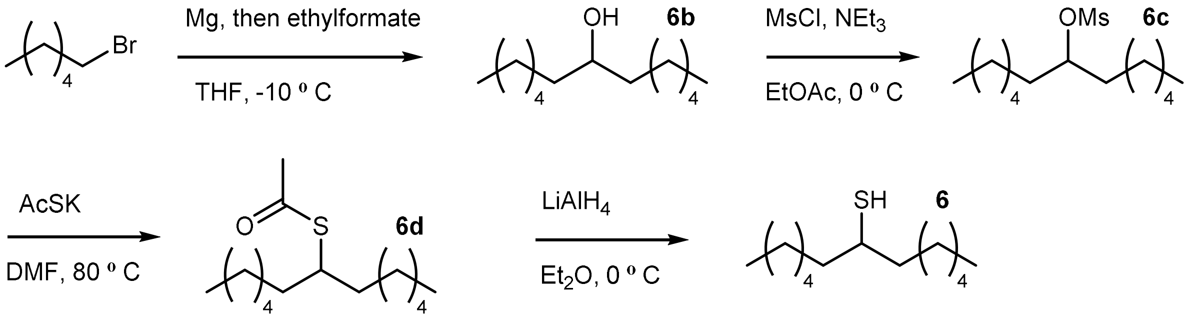

Scheme S6.1: Synthesis route for the preparation of compound **6**.

<u>Synthesis of 7-tridecanol (**6b**):</u>

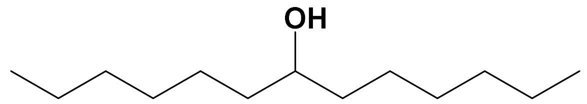

A solution of 1-bromohexane (16.8 mL, 120 mmol) in dry THF (200 mL) was cooled to -10 ˚C, after which magnesium turnings (2.92 g, 120 mmol) were added portionwise over one hour. A solution of ethyl formate (4.7 mL, 54 mmol) in THF was added dropwise, while maintaining the temperature below 10 ˚C. Next, the reaction mixture was allowed to warm to room temperature at which it was stirred for 6 hours. The reaction was quenched with saturated NH_4Cl and then acidified using 1 M HCl. The product was extracted using diethyl ether (3x50 mL) and the combined extracts were washed with brine and dried using $MgSO_4$. After removal of the solvent, the crude product was purified using flash chromatography (7% EtOAc in hexane) which yielded (after solvent removal) 7-tridecanol as colourless crystals. NMR spectra were in agreement with literature.[2]

^{1}H NMR (400 MHz, $CDCl_3$) δ 3.67 – 3.57 (m, 1H, C*H*OH), 1.52 – 1.35 (m, 4H, alk-CH_2), 1.35 – 1.17 (m, 16H, alk-CH_2), 0.87 (t, *J* = 7.0 Hz, 6H, CH2CH_3).
^{13}C NMR (101 MHz, $CDCl_3$) δ 72.2 (*C*OH), 37.8 (*C*H_2COH), 32.0 (alk-*C*H_2), 29.5 (alk-*C*H_2), 25.8 (alk-*C*H_2), 22.8 (alk-*C*H_2), 14.4 (CH_2*C*H_3).

<u>Synthesis of tridecan-7-yl methanesulfonate (**6c**):</u>

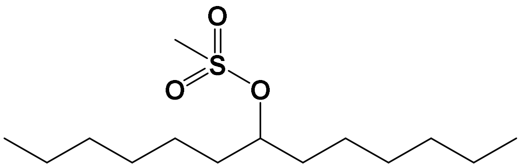

Triethylamine (8.25 mL, 60 mmol) and 7-tridecanol were added to ethyl acetate (100 mL) and cooled to 0 ˚C. Methanesulfonyl chloride (4.8 mL, 60 mmol) was added slowly, and the resulting suspension was stirred for one hour. Dilute hydrochloric acid was added (1M, 100 mL), after which the organic layer was extracted, washed with brine and dried using $MgSO_4$. Solvent removal yielded tridecan-7-yl methanesulfonate as a colourless oil (14.5 g, 96% over two steps).

^{1}H NMR (400 MHz, $CDCl_3$) δ 4.70 (quintet, 1H, C*H*OMs, *J* = 6.5 Hz), 2.99 (s, 3H, $S(O)_2OCH_3$) 1.73 – 1.64 (m, 4H, alk-CH_2), 1.46 – 1.23 (m, 16H, alk-CH_2), 0.88 (t, *J* = 7.0 Hz, 6H, CH_2CH_3).
^{13}C NMR (101 MHz, $CDCl_3$) δ 84.5 (*C*OMs), 38.9 ($CHOSO_2CH_3$), 34.6 (CH_2COH), 31.8 (alk-CH_2), 29.2 (alk-CH_2), 25.1 (alk-CH_2), 22.7 (alk-CH_2), 14.2 (CH_2CH_3).

S-(tridecan-7-yl) ethanethioate (**6d**):

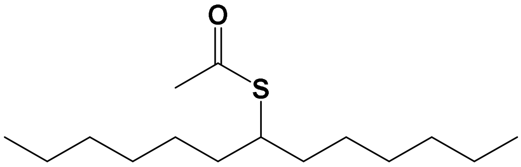

A solution of tridecan-7-yl methanesulfonate (14.5 g, 52 mmol) and potassium thioacetate (11.8 g, 104 mmol) in DMF (200 mL) was heated to 80 ˚C resulting in the formation of a dark red gelatinous solution. After heating for 5 hours, the reaction was cooled to room temperature, after which brine (150 mL) and diethyl ether (150 mL) were added. The organic layer was washed with brine (2x200 mL), dried with $MgSO_4$. After removal of the solvent, the crude product was purified using flash chromatography (5% EtOAc in hexane) to yield S-(tridecan-7-yl) ethanethioate as a dark red oil (6.31 g, 47%).

^{1}H NMR (400 MHz, $CDCl_3$) δ 3.54 – 3.46 (tt, 1H, C*H*OAc, *J* = 5.5 Hz, 7.8 Hz), 2.31 (s, 3H, $SC(O)CH_3$) 1.64 – 1.45 (m, 4H, alk-CH_2), 1.42 – 1.21 (m, 16H, alk-CH_2), 0.88 (t, *J* = 7.0 Hz, 6H, CH_2CH_3).
^{13}C NMR (101 MHz, $CDCl_3$) δ 196.3 ($SC(O)CH_3$) 44.9 (*C*SAc), 35.0 (CH_2COH), 31.9 (alk-CH_2), 30.9 ($SC(O)CH_3$) 29.3 (alk-CH_2), 26.9 (alk-CH_2), 22.8 (alk-CH_2), 14.2 (CH_2CH_3).

7-tridecanethiol (**6**):

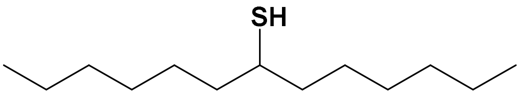

S-(tridecan-7-yl) ethanethioate (6.31 g,), dissolved in dry diethyl ether (20 mL) was added dropwise to a suspension of $LiAlH_4$ (1.98 g, 52 mmol) in dry diethyl ether (100 mL) at 0 ˚C. The mixture was allowed to warm to room temperature at which it was stirred for one hour. After cooling to 0 ˚C, the excess of $LiAlH_4$ was quenched using saturated NH_4Cl, after which the solution was acidified to pH 1 using 3 M HCl. The organic layer was extracted (2x50 mL distilled water), washed with brine (50 mL) and dried using $MgSO_4$. Purification using flash chromatography (100% hexanes, R_f = 0.7) yielded 7-tridecanethiol as a pale tan oil (4.37 g, 83%).

^{1}H NMR (400 MHz, CDCl3) δ 2.83 – 2.73 (m, 1H, C*H*SH), 1.66 – 1.58 (m, 2H, C*H*CHSH), 1.52 – 1.41 (m, 4H, alk-CH_2), 1.38 – 1.19 (m, 14H, alk-CH_2), 0.89 (t, 6H, CH_2CH_3, *J* = 7.0 Hz)
^{13}C NMR (101 MHz, $CDCl_3$) δ 41.4 (*C*SH), 39.2 (CH_2CSH), 31.9 (alk-CH_2), 29.2 (alk-CH_2), 27.2 (alk-CH_2), 22.8 (alk-CH_2), 14.2 (CH_2CH_3).
vmax / cm-1 2956, 2923, 2855, 2160, 2027, 1465, 1378, 725
EI MS found m / z [M]+ 216.1966, $C_{13}H_{28}S$ calculated 216.1906.

2-nitro-5-(tridecan-7-yldisulfaneyl)benzoic acid (**7**):

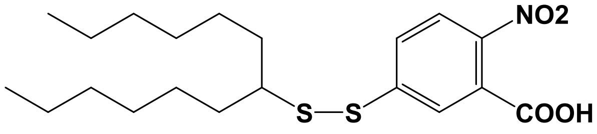

5,5’-Dithiobis-(2-nitrobenzoic acid) (332 mg, 0.84 mmol) and trimethylamine (0.36 mL, 0.84 mmol) were dissolved in 4 mL DCM. To this solution was added 7-tridecanethiol (200 mg, 0.92 mmol, 1.1 equiv.). The mixture was stirred for one hour, after which 20 mL of a 1M HCl solution was added. The aqeous layer was extracted with DCM, the organic fractions combined and dried using $MgSO_4$. After removal of the

solvent, the crude product was purified using flash chromatography (2% AcOH in DCM), yielding 2-nitro-5-(tridecan-7-yldisulfaneyl)benzoic acid as a light yellow oil (220 mg, 63% yield)

^{1}H NMR (400 MHz, $CDCl_3$) δ 10.39 (s, 1H, CO_2*H*), 7.99 (d, *J* = 2.0 Hz, 1H, C*H*CNO_2), 7.92 (d, *J* = 8.5 Hz, 1H, SCC*H*CO_2H), 7.82 (dd, *J* = 8.5, 2.0 Hz, 1H, SCC*H*CH), 2.81 (p, *J* = 6.5 Hz, 1H, C*H*S), 1.62 (q, *J* = 7.5 Hz, 4H, CH_2CHS), 1.50 – 1.36 (m, 4H, alk-CH_2), 1.31 – 1.24 (m, 12H, alk-CH_2), 0.90 (t, *J* = 7.0 Hz, 6H, CH_2CH_3).
^{13}C NMR (101 MHz, $CDCl_3$) δ 170.5 (*C*O_2H), 147.0 (*C*NO_2), 145.8 (*C*SSCHCH_2), 128.8 (*C*CO_2H), 127.1 (*C*CNO_2), 126.8 (CSS*C*CO_2H), 124.5 (*C*CSSCHCH_2), 53.6 (*C*HSS), 33.8 (*C*H_2CHSS), 31.6 (alk-*C*H_2), 29.1 (alk-*C*H_2), 26.7 (alk-*C*H_2), 22.6 (alk-*C*H_2), 14.0 (CH_2*C*H_3).
vmax / cm^{-1}: 2955, 2926, 2855, 1707, 1566, 1519,1466, 1416, 1340, 1289, 1145, 1107, 905, 872, 846, 754, 720;
HRMS *m / z* found [M-H]- 412.16225, $C_{20}H_{30}O_4NS_2$ calculated 412.16217.

7) UPLC calibration:

The concentration of UV active components of the reaction were monitored using a Waters Acquity ultra performance liquid chromatography UPLC H-Class system with photodiode array (PDA) detector. Instrument control and data processing were performed using Empower software. An Acquity UPLC BEH C18 column (130 Å, 1.7 µm, 2.1mm× 50 mm) was used. A mixture of H2O:MeCN:TFA in H2O with a gradient of 93:2:5 → 0:95:5 over 5 min was used as mobile phase. Peak areas were integrated at a wavelength of 330 nm. Solutions containing the different species were prepared in TRIS buffer (0.5 M, pH 9.00). Each point is the average of three measurements of the same sample. Error bars for the standard deviation of each point are plotted but are too small for observation. Buffer preparation has been previously described by Morrow et al.[3]

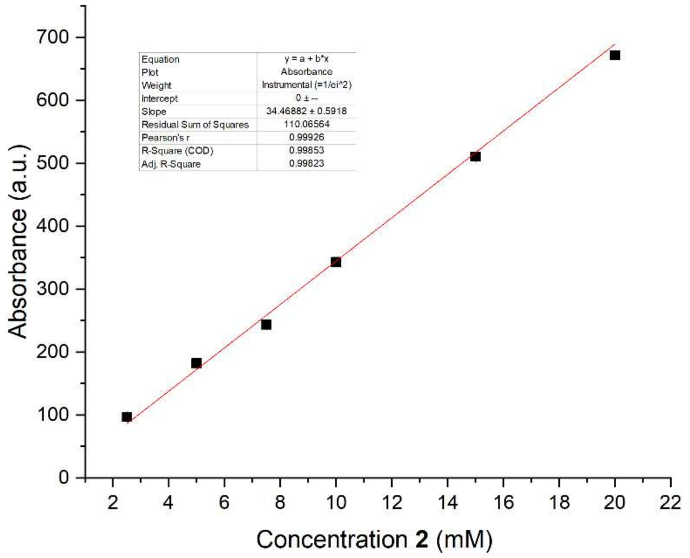

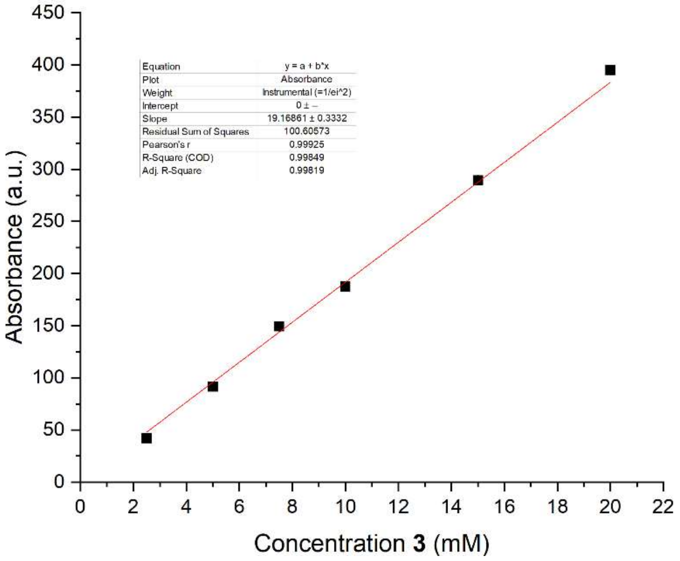

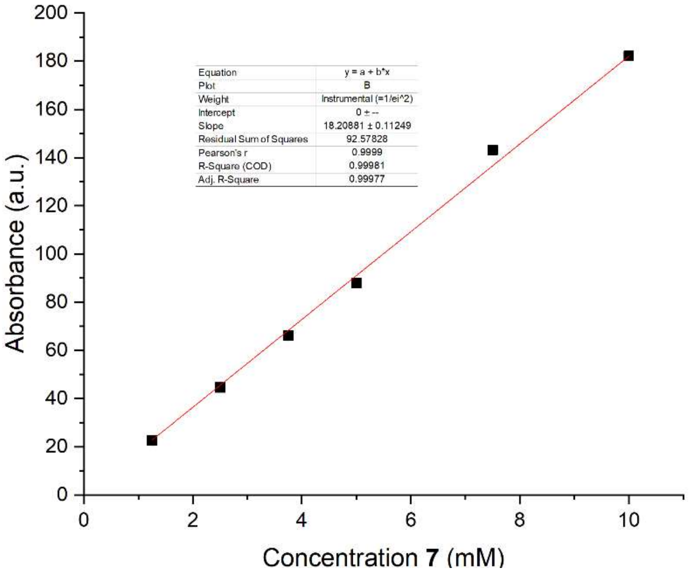

8) NMR spectra:

7-tridecanethiol **(6b)**:

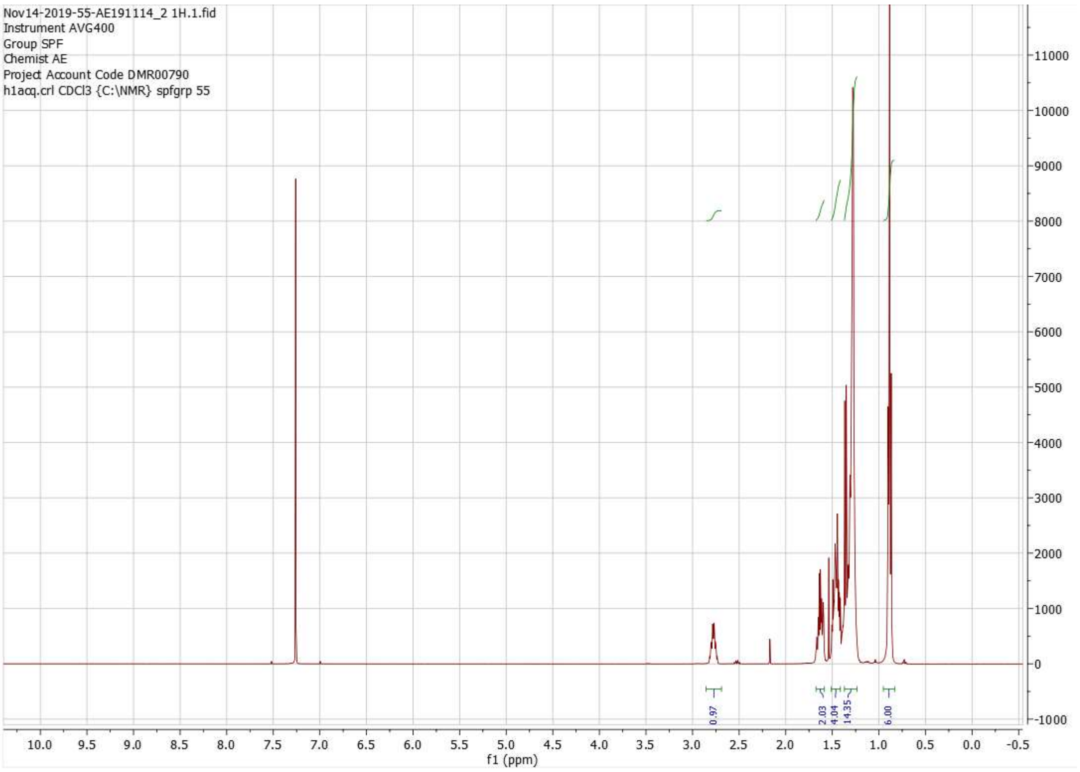

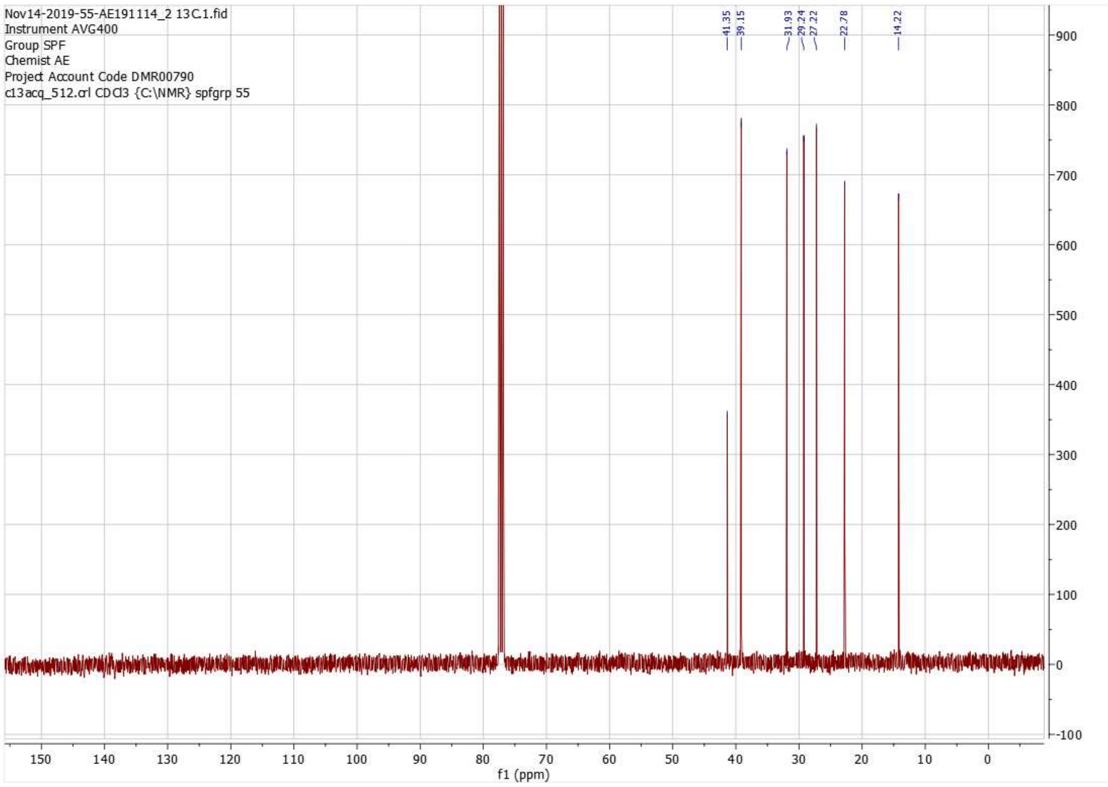

tridecan-7-yl methanesulfonate (**6c**)

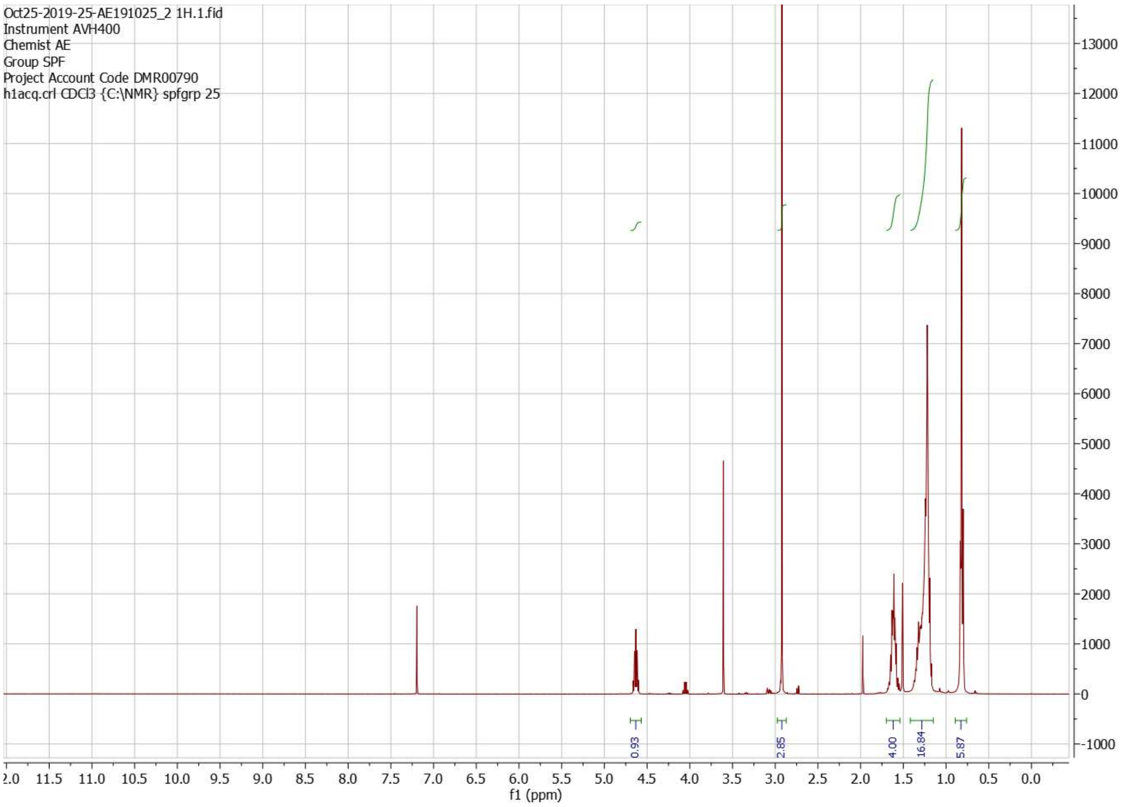

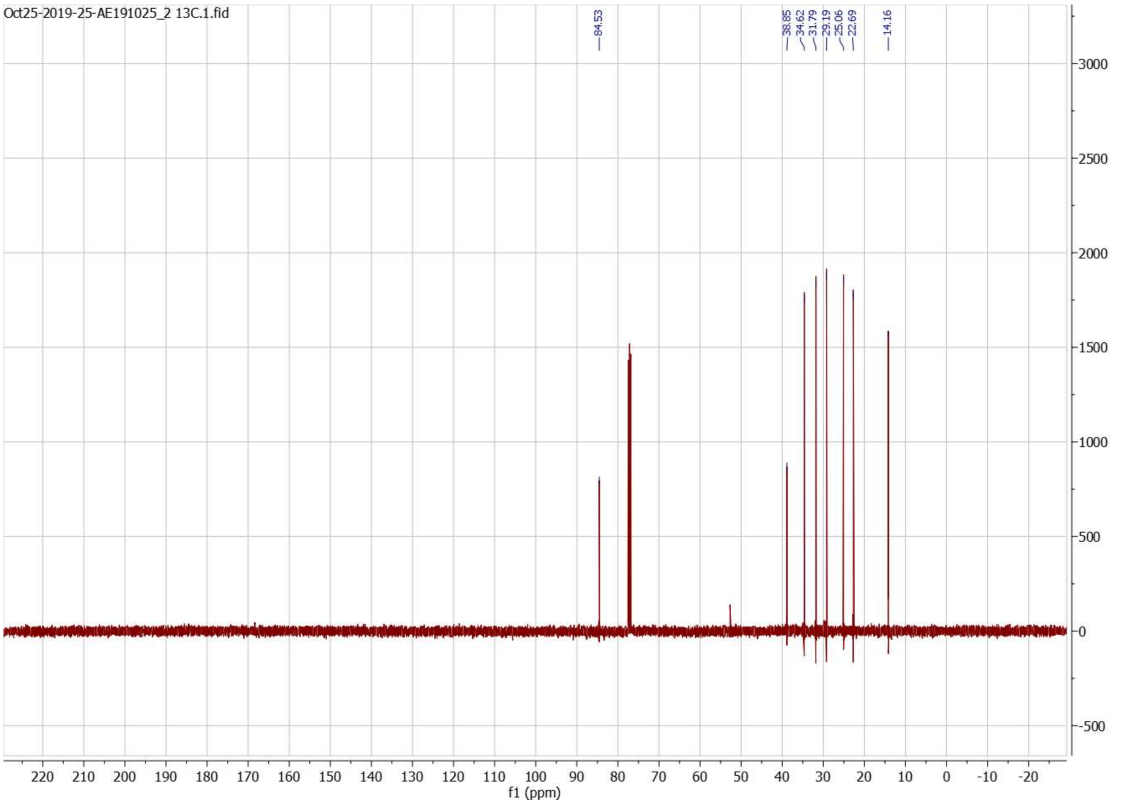

S-(tridecan-7-yl) ethanethioate **(6d)**:

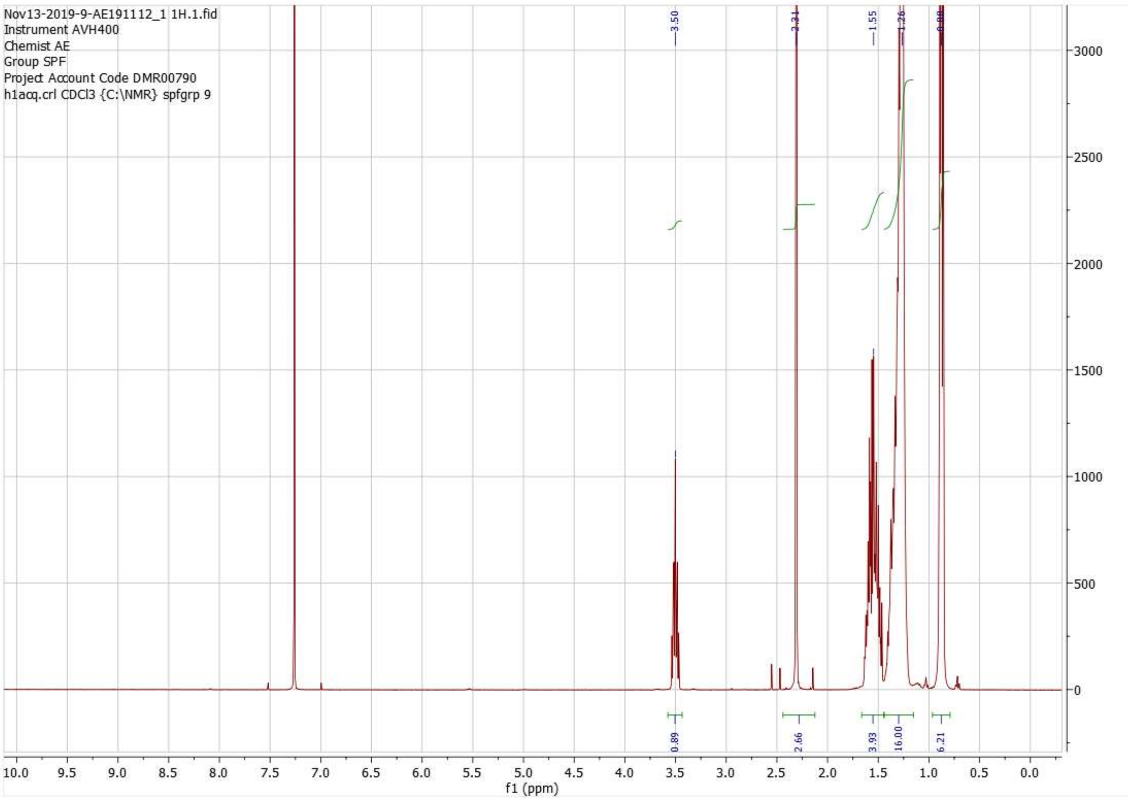

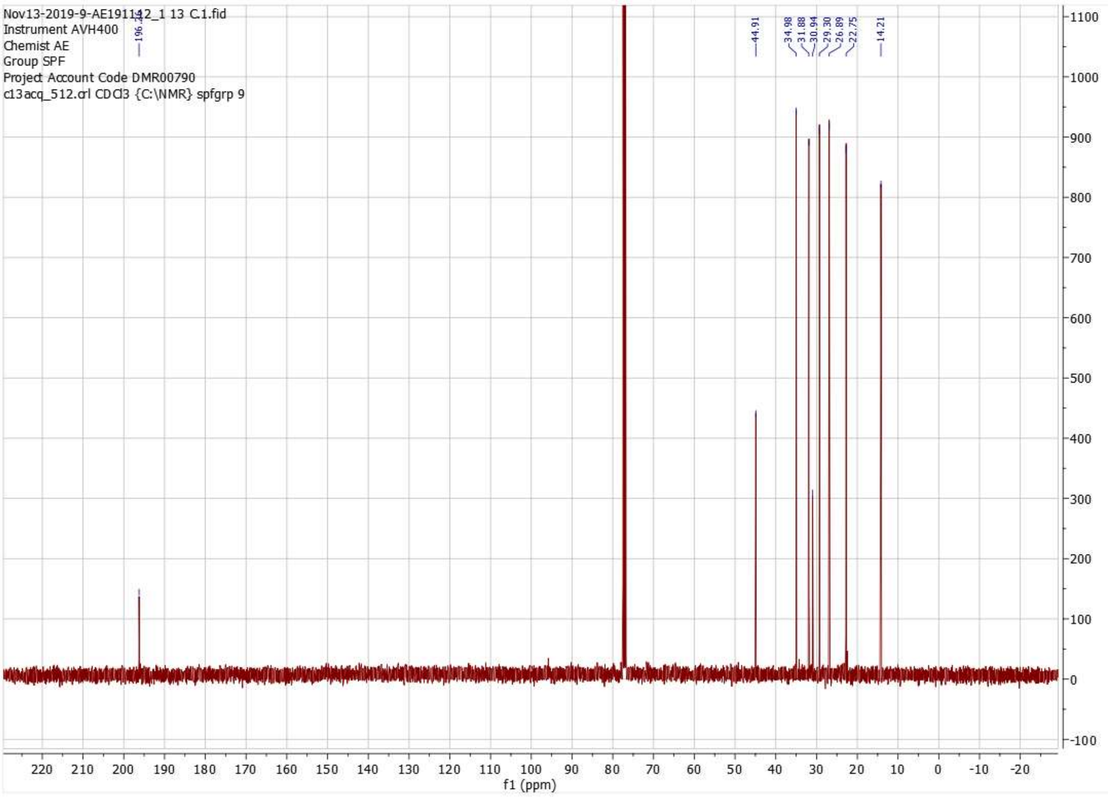

7-tridecanethiol (**6**):

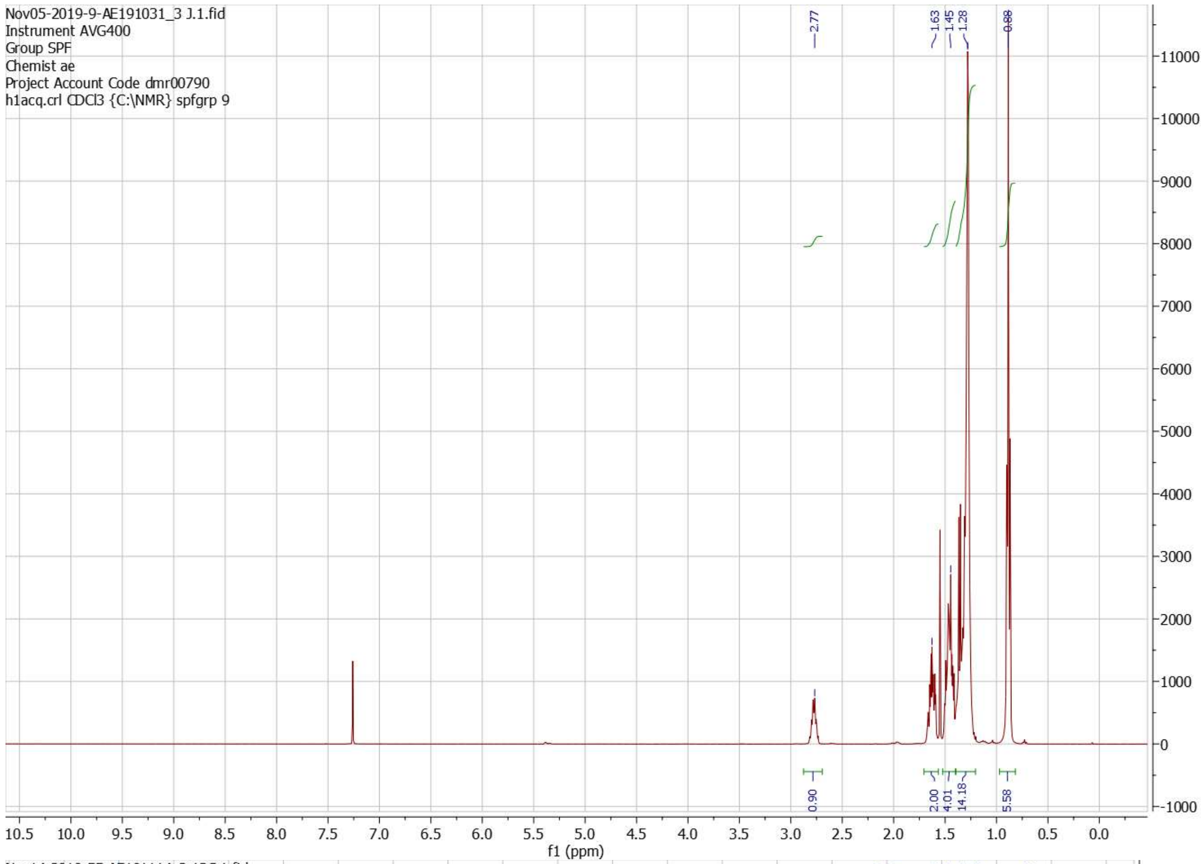

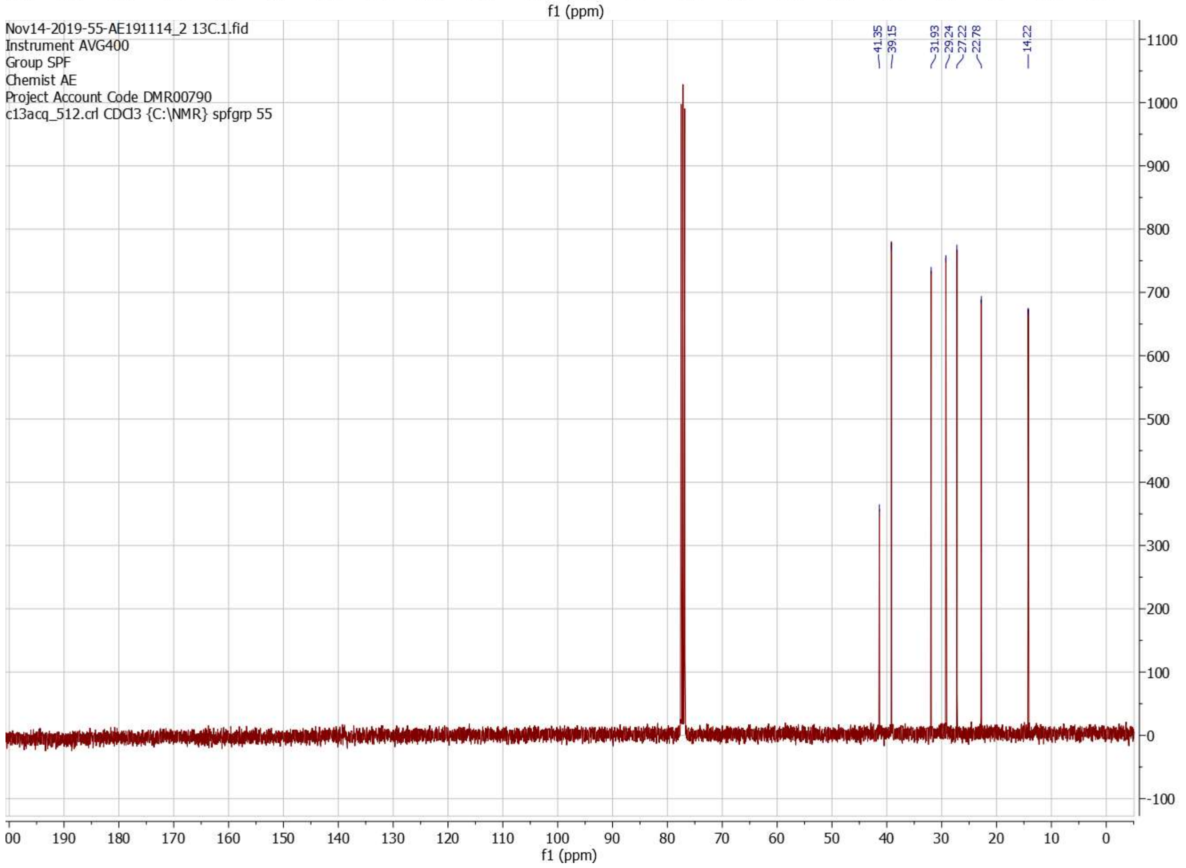

2-nitro-5-(tridecan-7-yldisulfaneyl)benzoic acid (**7**):

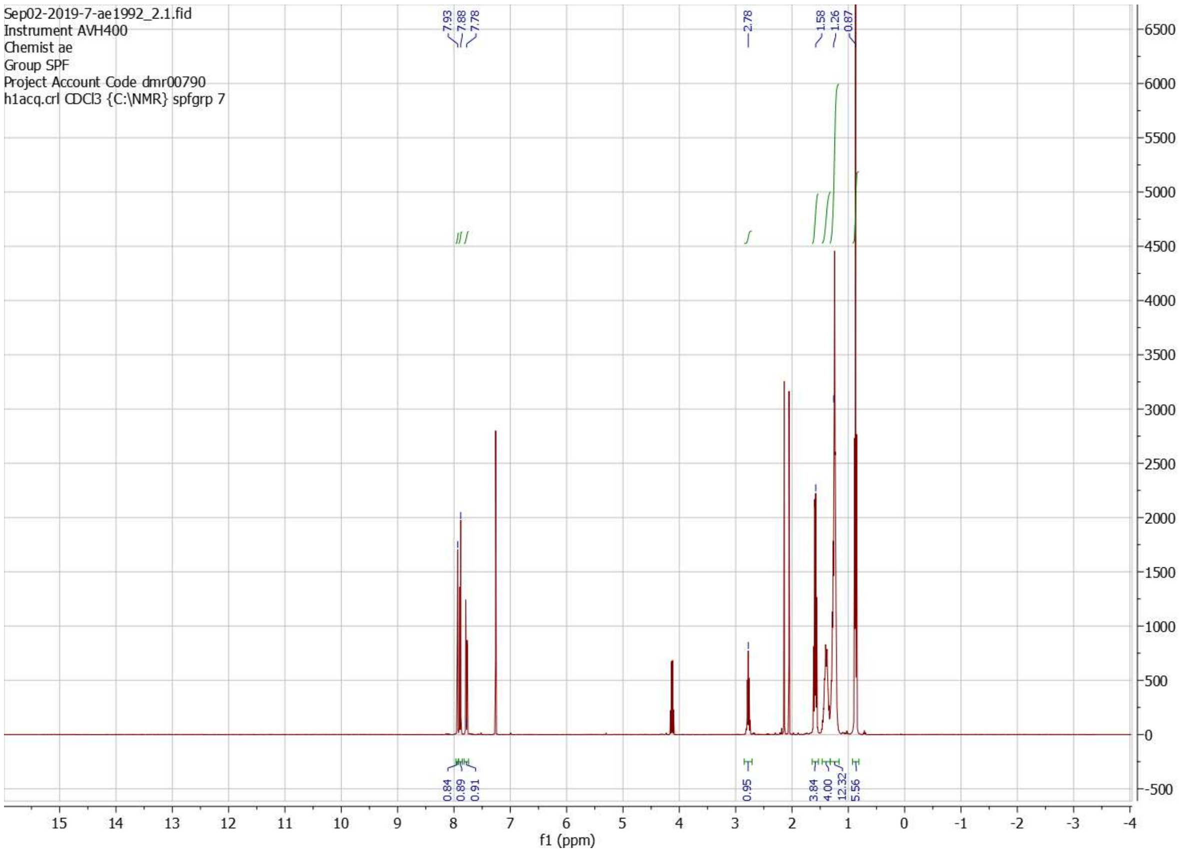

9) References:

1. Young, G.; Hundt, N.; Cole, D.; Fineberg, A.; Andrecka, J.; Tyler, A.; Olerinyova, A.; Ansari, A.; Marklund, E. G.; Collier, M. P.; Chandler, S. A.; Tkachenko, O.; Allen, J.; Crispin, M.; Billington, N.; Takagi, Y.; Sellers, J. R.; Eichmann, C.; Selenko, P.; Frey, L.; Riek, R.; Galpin, M. R.; Struwe, W. B.; Benesch, J. L. P.; Kukura, P., Quantitative mass imaging of single biological macromolecules. *Science* **2018,** *360* (6387), 423-427.
2. Yue, W.; Zhao, Y.; Shao, S. Y.; Tian, H. K.; Xie, Z. Y.; Geng, Y. H.; Wang, F. S., Novel NIR-absorbing conjugated polymers for efficient polymer solar cells: effect of alkyl chain length on device performance. *J. Mater. Chem.* **2009,** *19* (15), 2199-2206.
3. Morrow, S. M.; Colomer, I.; Fletcher, S. P., A chemically fuelled self-replicator. *Nat. Commun.* **2019,** *10*.
